# Gynecological cancer awareness and attitudes toward cancer screening: A descriptive and correlational study among nurses

**DOI:** 10.12669/pjms.41.10.12112

**Published:** 2025-10

**Authors:** Pinar Uzunkaya Oztoprak, Pelin Calpbinici

**Affiliations:** 1 Pinar Uzunkaya Oztoprak Obstetrics and Gynecology Nursing Department, Faculty of Nursing, Hacettepe University, 06080, Ankara, Turkiye; 2 Pelin Calpbinici Department of Nursing, Obstetrics, Women’s Health and Disease Nursing, Semra and Vefa Faculty of Health Sciences, Nevsehir Haci Bektas Veli University, 50040, Nevsehir, Turkiye

**Keywords:** Awareness, Cancer screening, Gynecological cancer, Nursing

## Abstract

**Objective:**

This study aimed to determine nurses’ awareness of gynecological cancer, identify influencing factors, and assess their attitudes toward cancer screening.

**Methods:**

A cross-sectional, descriptive, and correlational study was conducted with 230 female nurses at a state hospital in Turkey between January to June 2024. Data were obtained using a Demographic Information Form, the Gynecological Cancer Awareness Scale (GCAS), and the Attitudes Towards Cancer Screening Scale (ATCS). Numbers, percentages, means, Pearson correlation coefficient, and multiple linear hierarchical regression analysis were used to evaluate the data.

**Results:**

The GCAS score of the nurses was 159.46±21.39, whereas the ATCS score was 76.51±14.03. The difference between the GCAS score and the ATCS score and the variables of age, working time in the profession, menopausal status, family history of cancer and gynecological cancer, and participation in cancer screening were found to be significant (p<0.05). The results of hierarchical regression analysis showed that socio-demographic variables (age, working time in the profession, etc.) and cancer-related characteristics (family history of gynecological cancer, cancer screening, etc.) and attitudes towards cancer screening were significant predictors of awareness of gynecological cancer.

**Conclusion:**

Nurses’ awareness of gynecological cancer was above average, and attitudes toward screening were moderate. However, nurses’ awareness and attitudes towards gynecological cancers were not at the desired level because they are professional members who can lead society in gynecological cancer awareness and cancer screening.

## INTRODUCTION

Gynecological cancers are among the leading causes of morbidity and mortality in women globally, second only to breast cancer.^1^ Cervical, endometrial, and ovarian cancers are the most prevalent types worldwide^1^ and in Türkiye.^2^ Despite their prevalence, these cancers are largely preventable and treatable when detected at an early stage through effective screening and awareness programs.^3^,^4^ Nurses play an essential role in the early detection and prevention of gynecological cancers. As frontline healthcare providers, they are uniquely positioned to educate women, identify high-risk groups, recognize early symptoms, and encourage participation in screening initiatives.^5^,^6^ Given that the nursing workforce is predominantly female, nurses must possess adequate knowledge and awareness not only to protect their own health but also to serve as advocates for public health within their communities.^3^,^7^

Raising awareness among nurses about gynecological cancers and their associated screening practices is crucial for enhancing preventive health strategies. Their engagement in screening programs can significantly influence societal attitudes and participation rates.^4^-^6^ Therefore, assessing nurses’ awareness and attitudes toward gynecological cancer screening is a critical step in identifying gaps and informing educational and policy interventions.

This study aimed to evaluate nurses’ awareness of gynecological cancer, identify influencing factors, and examine their attitudes toward cancer screening.

## METHODS

This cross-sectional study was conducted at a public hospital in Central Anatolia, Türkiye, between January-June 2024. Of 360 female nurses, 230 met inclusion criteria (female, aged 20-65, actively working, no psychiatric or gynecological illness). Exclusions (n=130) included leave or incomplete data. Post-hoc power (G*Power 3.1.9.7) showed 99% power (effect size=0.420, CI=95%).

### Ethical considerations:

The study was approved by the Non-Interventional Research Ethics Committee of the relevant university (Approval date: September 15, 2023; Decision number: 2023/05). Institutional permission was also obtained from the hospital (Approval date: January 9, 2023; Decision number: E26171210-929-233554941). All participants were informed about the purpose, benefits, and duration of the study and provided written informed consent

Three instruments were used for data collection:

### Demographic information form:

Developed by the researchers based on literature,^8^-^10^ this Form included 18 questions assessing sociodemographic characteristics and cancer-related information.

### Gynecological cancer awareness scale (GCAS):

This 41-item, 5-point Likert-type scale was developed by Alp Dal and Ertem^11^ to assess awareness of gynecological cancer among Turkish women aged 20-65. The scale consists of four subdimensions: Routine Check-ups, Risk Awareness, Prevention Awareness, and Early Diagnosis. The total score ranges from 41 to 205, with higher scores indicating greater awareness. In the original development study, Cronbach’s alpha was 0.94^11^; in the current study, it was 0.95.

### Attitudes toward cancer screening scale (ATCS):

Developed by Öztürk et al.^12^, this 24-item scale uses a 5-point Likert format to evaluate individuals’ attitudes toward cancer screening. Higher scores reflect more favorable attitudes. The Cronbach’s alpha in the original study was 0.96^12^, and 0.79 in this study.

### Data collection procedure:

Data were collected through face-to-face interviews. After providing information about the study, voluntary participants were given the data collection forms and asked to complete them within one day. The completed forms were then collected by the researchers.

### Statistical analysis:

Data were analyzed using SPSS version 25.0 (IBM Corp., Armonk, NY, USA). Descriptive statistics (frequencies, percentages, means, and standard deviations) were used to summarize the data. Pearson correlation coefficients were calculated to assess relationships between variables. Multiple hierarchical linear regression analysis was performed to identify predictors of gynecological cancer awareness. Assumptions of normality, multicollinearity, and autocorrelation were checked before conducting regression analyses. A p-value of <0.05 was considered statistically significant.

## RESULTS

The nurses had a mean age of 35.03±9.11 years; 40.9% were aged 21-30. Most were married (67%) and held at least a bachelor’s degree (86.6%). About 34.8% had 1-5 years of experience, 40% worked in internal units, 61.7% had children, 8.3% were menopausal, and 17% had chronic illnesses (Table-I). A significant difference was found between nurses’ mean age and the GCAS total score, routine control score, risk awareness score, and prevention awareness score (p<0.05). Nurses aged 41 and above scored higher than those aged 21-30. Experience also influenced scores; nurses with 21+ years of experience had higher scores than those with 1-5 years. Nurses with children had higher risk awareness scores, and those in menopause had higher scores in total, routine control, and prevention awareness. Nurses with chronic diseases had higher routine control scores (p<0.05) (Table-I).

**Table-I:** Comparison of nurses’ socio-demographic information and mean scores of nurse from GCAS and its subdimensions (n=230).

Characteristics	n	%	GCAS	Routine Control	Risk Awareness	Prevention Awareness	Early Diagnosis
***Age (years)***							
21-30 age^1^	90	39.1	154.05±14.66	85.26±10.33	30.35±4.43	21.74±3.18	16.68±2.66
31-40 age^2^	70	30.4	159.15±23.77	87.48±14.57	31.75±6.68	22.47±4.61	17.44±2.96
41 age and above^3^	70	30.4	166.71±24.17	92.45±14.26	32.94±7.27	23.60±4.14	17.71±2.75
F/p			7.286/0.001	6.174/0.002	3.571/0.030	4.334/0.014	2.965/0.054
Post hoc**			3>1	3>1	3>1	3>1	-
***Marital status***							
Married	154	67.0	160.68±23.23	88.61±14.27	32.01±6.62	22.75±4.29	17.30±2.89
Single	76	33.0	156.98±16.91	87.15±10.95	30.67±5.10	22.07±3.37	17.07±2.62
t/p			1.370/0.172	0.781/0.436	1.693/0.092	1.199/0.232	0.574/0.566
***Educational status***							
High School	10	4.3	169.300±22.75	92.80±13.75	33.40±7.05	25.30±3.74	17.80±2.34
Associate Degree	21	9.1	159.00±22.25	88.23±14.60	30.66±7.17	22.52±3.78	17.57±2.11
Undergraduate and Postgraduate	199	86.6	159.01±21.22	87.88±13.11	31.57±6.04	22.39±4.02	17.16±2.89
KW/p			1.386/0.500	1.206/0.547	0.895/0.639	4.445/0.108	0.326/0.849
***Working Time in the Profession***							
1-5 years	80	34.8	156.62±15.62	86.70±10.16	30.95±5.25	22.03±2.88	16.93±2.64
6-12 years	46	20.0	159.95±20.62	87.91±13.03	31.97±6.60	22.54±4.79	17.52±2.56
13-20 years	44	19.1	155.25±26.85	84.72±16.50	31.61±6.66	22.00±4.51	16.90±3.25
21 years and above	60	26.1	165.95±23.06	92.70±13.53	32.05±6.72	23.56±4.18	17.63±2.83
F/p			2.961/0.033	3.789/0.011	0.453/0.716	2.015/0.113	1.060/0.367
Post hoc*			4>1	4>1, 4>3			
***Worked Unit***							
Internal units	92	40.0	156.63±19.54	86.56±12.82	31.19±5.00	22.01±3.68	16.85±2.72
Surgical units	57	24.8	159.71±23.63	87.87±13.56	32.45±6.54	22.52±4.30	16.85±3.36
Emergency	39	17.0	159.46±17.32	88.25±10.97	30.15±7.05	23.33±3.30	17.71±2.03
Polyclinic	42	18.3	165.30±24.77	91.78±15.32	32.50±7.00	22.92±4.81	18.09±2.59
F/p			1.599/0.190	1.509/0.213	1.511/0.212	1.173/0.321	2.646/0.050
***Having a child***							
Yes	142	61.7	161.07±23.93	88.80±14.54	32.20±6.77	22.66±4.44	17.40±2.92
No	88	38.3	156.86±16.26	87.04±10.85	30.54±4.95	22.31±3.22	16.95±2.59
t/p			1.585/0.114	1.045/0.297	2.138/0.034	0.678/0.499	1.174/0.242
***Number of pregnancies***							
Nulligravida	81	35.2	157.16±16.72	87.14±11.18	30.70±4.96	22.32±3.34	16.98±2.67
Primigravida	42	18.3	156.21±16.17	85.50±10.60	31.40±6.57	21.73±3.66	17.57±2.15
Multigravida	107	46.5	162.47±25.66	89.90±15.34	32.28±6.79	23.00±4.54	17.28±3.11
F/p			2.034/0.133	2.026/0.134	1.542/0.216	1.668/0.191	0.628/0.535
***Menopausal status***							
Yes	19	8.3	173.31±19.64	97.84±9.45	33.78±7.22	24.52±4.06	17.15±3.09
No	211	91.7	158.21±21.13	87.25±13.22	21.36±6.06	22.35±3.97	17.23±2.78
t/p			2.999/0.003	3.409/0.001	1.640/0.102	2.282/0.023	-0.117/0.907
***Chronic disease status***							
Yes	39	17.0	163.28±19.52	92.20±11.13	30.71±5.99	23.05±4.32	17.30±3.03
No	191	83.0	158.68±21.71	87.29±13.52	31.74±6.22	22.42±3.95	17.21±2.76
t/p			1.226/0.222	2.122/0.035	0.877/0.346	0.148/0.375	0.348/0.851

***Abbreviation:*** GCAS= Gynecological cancer awareness scale, t= Independent t test, F= One way ANOVA, p < 0.05, Age (Mean ± SD)=35.03±9.11,

* Bonferroni test was used for multiple comparisons.

Of the nurses, 40.9% had a family member diagnosed with cancer, and 8.7% had a family member with gynecological cancer. While 61.7% had never undergone screening, Pap smear and mammography were the most used methods. Common reasons for not screening included perceived lack of need, fear of exams, and not being sexually active (Table-II).

**Table-II:** Comparison of nurses’ characteristics related to cancer and mean scores of nurse from GCAS and its subdimensions (n=230).

Characteristics	n	%	GCAS	Routine Control	Risk Awareness	Prevention Awareness	Early Diagnosis
***Presence of cancer in the family***							
Yes	94	40.9	163.84±21.47	90.98±12.83	32.22±6.28	23.10±4.25	17.52±2.72
No	136	59.1	156.43±20.87	86.115±13.23	31.11±6.09	22.13±3.80	17.02±2.85
t/p			2.614/0.010	2.757/0.006	1.335/0.183	1.780/0.077	1.308/0.192
***Gynecological cancer in the family***							
Yes	20	8.7	182.50±15.93	101.00±7.67	35.80±7.87	26.50±3.05	19.20±1.32
No	210	91.3	157.26±20.55	86.90±13.03	31.16±5.86	22.15±3.89	17.04±2.84
t/p			5.335/<0.001	4.751/<0.001	2.566/0.018	5.927/<0.001	6.082/<0.001
***Regular gynecological exams***							
Yes	30	13.0	167.60±29.75	91.50±17.03	33.13±8.49	25.16±4.74	17.80±3.63
No	200	87.0	158.24±19.64	87.62±12.57	31.33±5.75	22.13±3.75	17.14±2.66
t/p			2.225/0.025	1.198/0.239	1.489/0.138	3.343/0.002	0.950/0.349
***Cancer screening status***							
Yes	88	38.3	163.87±22.30	90.59±14.24	31.95±6.58	23.31±4.37	18.01±2.50
No	142	61.7	156.72±20.40	86.60±12.42	31.33±5.92	22.04±3.70	16.74±2.88
t/p			2.492/0.013	2.234/0.026	2.365/0.019	2.275/0.024	3.511/0.001
***Pap-smear test having***							
Yes	72	31.3	164.26±20.99	90.77±13.44	32.02±6.41	23.40±4.06	18.05±2.28
No	158	68.7	157.27±21.27	86.92±13.03	31.36±6.08	22.13±3.94	16.85±2.94
t/p			2.321/0.021	2.058/0.041	0.758/0.449	2.243/0.026	3.064/0.002
***Mammography test having***							
Yes	44	19.1	165.02±25.97	92.45±16.26	31.25±6.82	23.43±4.61	17.88±2.97
No	186	80.9	158.14±20.00	87.10±12.27	31.64±6.03	22.31±3.84	17.07±2.75
t/p			1.929/0.055	2.047/0.045	-0.380/0.704	1.661/0.098	1.731/0.085
***Colonoscopy test having***							
Yes	12	5.2	161.66±26.55	87.58±18.76	32.75±6.13	23.00±3.78	18.33±1.92
No	218	94.8	159.33±21.13	88.16±12.95	31.50±6.19	22.50±4.03	17.16±2.83
Z/p			-0.831/0.406	-0.459/0.646	-0.911/0.362	-0.702/0.482	-1.372/0.170
***Endoscopy test having***							
Yes	13	5.7	160.07±27.66	87.38±18.30	32.07±6.25	22.38±4.55	18.23±2.12
No	217	94.3	159.42±21.03	88.17±12.95	31.53±6.19	22.53±3.99	17.17±2.83
Z/p			-0.367/0.714	-0.384/0.701	-0.439/0.661	-0.103/0.918	-1.328/0.184
***Reasons for not getting cancer screening (n=142)***							
Not Feeling the Need	47	33.1	156.23±14.96	86.82±10.23	31.36±4.65	21.70±3.15	16.34±2.42
Afraid of Gynecological Examination	38	26.8	161.89±18.89	89.76±10.44	32.31±6.16	23.02±3.73	16.78±2.59
Not Seeing Oneself at Risk	8	5.6	151.00±12.96	82.50±9.03	30.87±2.94	21.50±2.72	16.12±2.85
Thinking of Getting it in the Future	21	14.8	155.38±22.63	84.23±12.07	31.14±7.48	22.28±4.33	17.71±3.24
Not Being Sexually Active	18	12.7	157.00±20.76	86.94±13.55	31.44±6.67	21.22±3.38	17.38±2.72
No Complaints	10	7.0	146.30±39.48	81.20±24.43	28.00±7.19	21.30±5.57	15.80±4.87
KW/p			2.395/0.663	4.081/0.395	1.063/0.900	3.218/0.522	7.939/0.094

***Abbreviation:*** GCAS= Gynecological cancer awareness scale, t= Independent t test, KW= Kruskal Wallis test, Z= Mann Whitney U test, p<0.05 r: Pearson’s correlation, SD: standard deviation, r<0.2 very weak relationship, r=0.2-0.4 weak relationship between, r=0.4-0.6 medium relationship between.

Nurses with a family history of gynecological cancer or prior cancer screening had higher GCAS scores. Those undergoing regular gynecological exams or mammography also scored higher. Pap smear was linked to higher total, routine control, prevention, and early diagnosis scores (Table-II). Additionally, positive correlations were found between ATCS and the GSAC total score, risk awareness, and prevention awareness (Table-III).

**Table-III:** Relationship between nurses’ gynecological cancer awareness and their attitudes towards cancer screening.

Scales	Mean± SD	ATCS (Mean± SD)	
		76.51±14.03	
		r	p
GCAS (Total)	159.46±21.39	0.177	0.007
Routine Control	88.13±13.26	0.087	0.189
Risk Awareness	31.56±6.18	0.311	<0.001
Prevention Awareness	22.53±4.01	0.154	0.020
Early Diagnosis	17.23±2.81	0.033	0.615

***Abbreviation:*** ATCS=Attitudes toward cancer screening scale, GCAS= Gynecological cancer awareness scale.

The results of the multiple linear hierarchical regression analysis conducted to examine the variables predicting nurses’ awareness of gynecological cancer are presented in Table-IV. Sociodemographic variables (age, working time in the profession, menopausal status) that were found to be significant in the initial stage were included in the model. In the subsequent stage, cancer-related characteristics (cancer in the family, gynecological cancer in the family, regular gynecological exam, previous cancer screenings, pap-smear test) were added to the model.

**Table-IV:** Hierarchical regression analysis of variables affecting nurses’ gynecological cancer awareness.

Independent Variables	Model 1			Model 2		Model 3	
	B	SE	β	B	SE	β	B	SE	β
Age	14.153	3.562	0.550*	11.534	3.478	0.448*	11.512	3.453	0.447*
Working time in the profession	-7.181	2.456	-0.405*	-6.871	2.417	-0.388*	-6.686	2.400	-0.377*
Menopausal status^1^	-10.717	5.336	-0.138*	-11.697	5.159	-0.151*	-10.820	5.139	-0.140*
Cancer in the Family 1				-3.046	2.739	-0.070	-3.232	2.721	-0.074
Gynecological Cancer in the Family^1^				-20.407	4.987	-0.269*	-19.988	4.955	-0.264*
Regular Gynecological Exams^1^				-1.185	4.274	-0.019	0.002	4.281	0.000
Cancer Screening^1^				3.114	5.505	0.071	2.683	5.468	-0.061
Pap-Smear^1^				-6.666	5.437	-0.145	-5.434	5.429	-0.118
ATCS							0.205	0.093	0.135*
F	8.841			6.907			6.707		
p	<0.001			<0.001			<0.001		
R^2^	0.105			0.200			0.215		
ΔR^2^	0.093			0.171			0.183		

Abbreviation: ATCS=Attitudes toward cancer screening scale,

* p<0.05; Durbin-Watson=1.705; Working time in the profession=21 years and above; 1=yes; Dependent variable: GCAS; SE: Standard Error.

Finally, in the third step, the variable attitude towards cancer screening was introduced into the model. The analysis revealed that among the sociodemographic variables in the first model, age (β = 0.550; p < 0.05), working time in the profession (β = -0.405; p < 0.05), and menopausal status (β = -0.138; p < 0.05) significantly predicted awareness of gynecological cancer, explaining 9.3% of the variance. Consequently, it was observed that age had a positive impact on awareness of gynecological cancer, while working time in the profession and menopausal status had a negative impact. Awareness of gynecological cancer was found to increase with age, and working time in the profession, and was higher among nurses who had undergone menopause. In the second model, the inclusion of variables related to nurses’ cancer characteristics explained 17.1% of the variance (F=6.907; p<0.001). It was noted that a family history of gynecological cancer (β=0.269; p<0.05) positively predicted awareness of gynecological cancer; thus, nurses with a family history of gynecological cancer exhibited higher awareness. Finally, in the third model, the addition of the variable attitude towards screening accounted for 18.3% of the variance, indicating that a more positive attitude towards cancer screening led to increased awareness of gynecological cancer (β = 0.135; p<0.05) (Table-IV).

## DISCUSSION

This study explored nurses’ awareness of gynecological cancer, identified factors that influence their awareness, and assessed their attitudes towards cancer screening. Our study found that nurses had an average score of 159.46±21.39 on the gynecological cancer awareness scale, indicating above-average awareness. Similarly, Alp Dal et al.^13^ reported a mean score of 159.96±24.27 among female health workers, with no difference between academicians, administrators, and health workers. Although no studies specifically on nurses were found, similar scores were reported among housewives and less-educated women, showing above-moderate awareness.^14^-^16^ A major strength of this study is that, unlike most research focusing on the general female population, it provides the first evidence on gynecological cancer awareness and screening attitudes among nurses in Turkey. Given that nurses are central to health education and cancer prevention, these findings offer valuable insights for clinical practice and public health. While nurses’ awareness appears comparable to that of the general population, their role in public education highlights the need for further improvement.

Previous studies have shown that gynecological cancer awareness is associated with sociodemographic factors such as age.^6^,^17^,^18^ In this study, nurses aged 41 and above were found to have higher awareness compared to those aged 21-30. Similarly, studies by Şenol Kaya et al.^19^ and Tekbaş^20^ reported higher awareness among women aged 36-50 and 45-50, respectively. Research by Teskereci et al.^14^, Öztürk et al.^17^, and Silveira et al.^21^ also supports the increase in knowledge, attitudes, and practices with age. These findings suggest that age-related cancer risk and perceived health status positively affect awareness, and they underscore the need for longitudinal research to track changes in awareness across the lifespan.

Nurses with ≥21 years of professional experience had significantly higher awareness than those with 1–5 years. Prior studies confirm that professional experience significantly influences awareness.^8^,^19^ This may result from increased patient exposure, interdisciplinary communication, and cumulative clinical experience. As experience grows, awareness likely increases due to greater encounters with gynecological conditions and perceived personal risk. These findings indicate that early-career nurses may require targeted training, and future intervention studies should assess whether structured educational programs improve awareness and screening behaviors among less-experienced staff.

Menopause increases the risk and incidence of gynecological cancers, making awareness crucial.^3^,^8^,^17^ Our study found higher awareness among menopausal nurses, consistent with previous research. This may be linked to age-related cancer risk, anxiety, and greater information-seeking behavior. Additionally, family history, especially of genetically linked cancers like ovarian and endometrial, is a major risk factor.^3^,^22^,^23^ Our study and others show that women with such history, including postmenopausal and reproductive-age groups, have higher gynecological cancer awareness—likely due to fear of inherited risk.^3^,^22^,^23^ Future studies could investigate how genetic counseling and family history awareness influence nurses’ preventive health practices, including their engagement in screening programs.

Nurses’ positive attitudes towards cancer screening, along with their high awareness of gynecological cancers, can positively impact societal attitudes.^5^,^7^ Our study found that nurses’ cancer screening attitude scores averaged 76.51±14.03, indicating a moderate level of attitudes. Bostan Akmeşe et al. and Altunbaş et al.^24^ reported slightly higher scores of 99.93±13.89. The moderate stance of nurses, crucial in raising public awareness, could be a significant factor contributing to the lower participation of Turkish women in cancer screening programs. This highlights a gap between knowledge and practice, suggesting that future studies should examine whether improved awareness among nurses translates into greater community participation in screening programs.

Regular gynecological exams and cancer screenings are crucial for the prevention of gynecological cancers.^3^,^7^ In our study, it was observed that as nurses’ attitudes toward cancer screening became more positive, their awareness levels increased. However, the participation of women in regular gynecological exams remains generally low.^25^ More than half of the nurses in our study reported not participating in cancer screenings due to a perceived lack of necessity, fear of gynecological exams, and not being sexually active. Previous studies have similarly highlighted that women often avoid regular gynecological exams due to reasons such as shame and fear.^4^,^5^,^7^ Nevertheless, women who undergo regular exams and cancer screenings tend to have higher awareness of gynecological cancers.^8^ This finding suggests that increasing awareness plays a significant role in enhancing participation in cancer screenings. Notably, nurses who are aware of the risk factors for gynecological cancers and take personal responsibility for their health proactively protect themselves and translate their awareness into behavior.

This is the first known study to assess gynecological cancer awareness and attitudes toward cancer screening among nurses in Turkey, offers significant clinical insights. Findings highlight a gap between nurses’ knowledge and practice, underscoring the need for targeted educational interventions. An increase in nurses’ awareness levels can enhance public participation in cancer screening programs.

### Limitations and Future Directions:

The single-center design may limit the generalizability of the findings; therefore, future multi-center studies encompassing diverse geographical regions and various types of hospitals are recommended. Additionally, longitudinal research is needed to examine how nurses’ knowledge and screening behaviors evolve over time. Intervention-based studies evaluating the effectiveness of targeted educational programs could further inform evidence-based strategies to enhance nurses’ competencies in gynecological cancer care. Addressing these areas in future research will not only strengthen the reliability of the evidence but also facilitate the development of more effective educational and policy initiatives.

## CONCLUSION

Nurses showed above-average awareness and moderate attitudes toward gynecological cancer screening. Awareness was influenced by certain socio-demographic and cancer-related factors. Positive attitudes were associated with higher awareness. Considering their important roles, nurses’ awareness and screening practices should be strengthened. Targeted education could enhance both personal behaviors and public guidance.

### Recommendations:

Training and awareness-based programs integrated into in-service training and undergraduate/graduate curricula can be recommended for increasing nurses’ awareness and fostering positive attitudes. Designing these trainings and programs in accordance with nurses’ sociodemographic and cancer-related characteristics may increase their effectiveness. Furthermore, it is recommended that the awareness and attitudes of male nurses regarding this issue be evaluated.

### Author`s Contribution:

**PUO:** Design of the study, writing and editing of the manuscript, critical review and final approval.

**PC:** Data collection, statistical analyses, assisted in editing the manuscript.

Both authors share equal responsibility for all aspects of the work and are responsible the integrity of the research.
